# The Study Protocol for the LINC (LUCAS in Cardiac Arrest) Study: a study comparing conventional adult out-of-hospital cardiopulmonary resuscitation with a concept with mechanical chest compressions and simultaneous defibrillation

**DOI:** 10.1186/1757-7241-21-5

**Published:** 2013-01-25

**Authors:** Sten Rubertsson, Johan Silfverstolpe, Liselott Rehn, Thomas Nyman, Rob Lichtveld, Rene Boomars, Wendy Bruins, Björn Ahlstedt, Helena Puggioli, Erik Lindgren, David Smekal, Gunnar Skoog, Robert Kastberg, Anna Lindblad, David Halliwell, Martyn Box, Fredrik Arnwald, Bjarne Madsen Hardig, Douglas Chamberlain, Johan Herlitz, Rolf Karlsten

**Affiliations:** 1 Department of Surgical Sciences/Anaesthesiology & Intensive Care, Uppsala University, Uppsala, Sweden; 2 Region Skånes Prehospitala Centrum, Skånes University Hospital, Lund, Sweden; 3 Regional Ambulance Service Utrecht, Utrecht, Netherlands; 4 Västerås central hospital, Västerås, Sweden; 5 Gävle center hospital, Gävle, Sweden; 6 South Western Ambulance Service NHS Foundation Trust, Abbey Court, Eagle way, Exeter, United Kingdom; 7 Physio-Control/Jolife AB, Ideon Science Park, 223 70, Lund, Sweden; 8 Institute of Primary Care and Public Health, Cardiff University School of Medicine, Cardiff, United Kingdom; 9 The Center of Prehospital Research in Western Sweden, University College of Borås, Borås, Sweden; 10 The Center of Prehospital Research in Western Sweden, Sahlgrenska University Hospital, Göteborg, Sweden

**Keywords:** Cardiac arrest, Mechanical chest compression, Defibrillation, External chest compressions, Ventricular fibrillation, Asystole, Pulseless electrical activity

## Abstract

**Background:**

The LUCAS™ device delivers mechanical chest compressions that have been shown in experimental studies to improve perfusion pressures to the brain and heart as well as augmenting cerebral blood flow and end tidal CO_2,_ compared with results from standard manual cardiopulmonary resuscitation (CPR). Two randomised pilot studies in out-of-hospital cardiac arrest patients have not shown improved outcome when compared with manual CPR. There remains evidence from small case series that the device can be potentially beneficial compared with manual chest compressions in specific situations. This multicentre study is designed to evaluate the efficacy and safety of mechanical chest compressions with the LUCAS™ device whilst allowing defibrillation during on-going CPR, and comparing the results with those of conventional resuscitation.

**Methods/design:**

This article describes the design and protocol of the LINC-study which is a randomised controlled multicentre study of 2500 out-of-hospital cardiac arrest patients. The study has been registered at ClinicalTrials.gov (http://clinicaltrials.gov/ct2/show/NCT00609778?term=LINC&rank=1).

**Results:**

Primary endpoint is four-hour survival after successful restoration of spontaneous circulation. The safety aspect is being evaluated by post mortem examinations in 300 patients that may reflect injuries from CPR.

**Conclusion:**

This large multicentre study will contribute to the evaluation of mechanical chest compression in CPR and specifically to the efficacy and safety of the LUCAS™ device when used in association with defibrillation during on-going CPR.

## Background

The LUCAS™ Chest Compression System (Physio-Control/Jolife AB, Lund, Sweden) has been in clinical use since 2003. Experimental data have shown improved perfusion pressures to the brain and heart, enhanced cerebral blood flow and higher end tidal CO_2_ as an indirect measure of cardiac output using the LUCAS™ device as compared with the effects of conventional manual CPR [1,2]. The LUCAS™ device has also shown higher end tidal CO_2_ values in out-of-hospital cardiac arrest (OHCA) victims compared with manual CPR [3]. There is some evidence from small case series that the device can be valuable in the catheterisation laboratory to facilitate Percutaneous Coronary Intervention (PCI) during cardiac arrest [4-6]. The potential benefit of the device has also been reported in special circumstances following accidental hypothermia/drowning and in some cases of in-hospital cardiac arrest [6-12]. This was recognised by the American and European guidelines for resuscitation in 2010 [13,14].

However, in a cluster randomised pilot study of OHCA patients, a comparison of manual versus mechanical chest compressions with the LUCAS™ device, using the ACLS guideline from 2005, did not show any outcome difference between the groups [15]. The study was performed in a 2-tier ambulance system with a 6 min delay from the start of treatment to application of LUCAS™ device and a substantial median interval (18 min) from estimated time of cardiac arrest. Defibrillation was not delivered during on-going mechanical chest compressions [15]. A second randomized pilot study in OHCA victims was performed 2005–2007 in Uppsala and Gävle, Sweden where the LUCAS™ device was used in combination with defibrillation during on-going chest compressions. It showed a trend to an increased return of spontaneous circulation (ROSC) rate (LUCAS = 40% vs. manual = 32%) despite interruptions for rhythm analysis and a median (± SD) of 13.1 ±7.2 min to start of mechanical chest compressions from estimated time of cardiac arrest [16].

The LINC-study was designed using the knowledge from previous studies, to evaluate the efficacy and safety of mechanical chest compressions using LUCAS™, in association with defibrillation during on-going CPR and with reduced delays. It is powered to detect superiority in four-hour survival of the modified resuscitation algorithm compared with conventional manual resuscitation in patients suffering from OHCA [13,14].

## Methods and design of the LINC-study

### Ethical approvals and study setting

The LINC-study is being conducted in accordance with regulatory requirements, Good Clinical Practices (GCP), and the ethical principles of the Declaration of Helsinki as adopted by the 18^th^ World Medical Assembly in Helsinki, Finland in 1964. The study was approved by the regional ethical review board in Uppsala, Sweden (Dnr. 2007/271), a research ethics committee in England (Dnr. 08/H0201/33), and the United Human Subjects Research Committees (VCMO) in the Netherlands (Dnr. NL 21034.10008. R-08.10E LINC). The study has been registered at ClinicalTrials.gov (http://clinicaltrials.gov/ct2/show/NCT00609778?term=LINC&rank=1) that supplies information for locating federal and privately supported clinical trials performed around the world.

Jolife AB, Lund, Sweden, the manufacturer of the LUCAS™ device, was the initial sponsor of the study, but since Jolife AB was acquired by Medtronic Inc., Minneapolis MN, USA (March 2011) and thereafter by Physio-Control, Redmond WA, USA the sponsorship was moved accordingly.

There are six participating Emergency Medical Services (EMS) in the LINC-study; Gävle, Malmö, Västerås and Uppsala in Sweden, Utrecht in the Netherlands, and Dorset in England. These centres could fulfill the following requirements: they have earlier experience of pre-hospital studies or in the use of LUCAS™; they are not participating in a clinical study that would be in conflict with the LINC-study; they have responders who are able to defibrillate manually rather than being restricted to the use of Automated External Defibrillators (AEDs); they enjoy good cooperation with their admitting hospitals; their median response interval is relatively brief; the number of patients who could be included was likely to be at least 40 patients/year but not more than 400 patients/year; the hospital to which their patients are admitted are able to perform PCI within 48 h following ROSC; and all surviving patients could be treated with hypothermia following ROSC, regardless of initial ECG rhythm unless contraindicated. Six more EMS was asked to participate but could not fulfill these criteria. Demographic data of the six participating EMS are presented in Table 1.

**Table 1:** Demographic data of the six participating EMS participating in the LINC-study

**Site**	**Primary investigator**	**Population**	**Start with LINC**	**Number of stations**	**Number of hospitals**	**Number of paramedics**	**Approx inclusions/year**	**LUCAS units**
Uppsala, Sweden	Sten Rubertsson	128.000	20080115	2	1	85	50	10
Gävle, Sweden	Robert Kastberg/Gunnar Skoog	127.000	20080115	4	1	106	55	9
Västerås, Sweden	Björn Ahlstedt	132.000	20080115	2	1	55	55	8
Malmö, Sweden	Johan Silfverstolpe	280.000	20081101	2	1	150	140	12
Dorset, England	Gillian Bryce/Dave Halliwell	400.000	20081210	5	2	100	135	26
Utrecht, The Netherlands	Rob Lichtveld	1.200.000	20081117	11	8	275	225	50

### Steering committee and contract research organisation (CRO)

The steering committee is monitoring the criteria for termination stated in the study protocol, in which the sponsor reserves the right to discontinue the study before recruitment of the intended number of subjects, but intends to exercise this right only for valid scientific or administrative reasons. These are: unexpectedly high proportion of adverse events; new findings about the investigational product that changes the benefit/risk ratio; the study protocol proves too difficult to achieve; recruitment of eligible subjects is far too slow; critical change in administrative or scientific standards at the sponsor organisation or at the participating organisations; or if the interim analyses show a difference between the groups with a p-value from a two-sided Fisher’s Exact Test of less than 0.005. The study has a steering committee that has the following members:

1. Sten Rubertsson MD PhD, Uppsala, Sweden (principal investigator)

2. Rolf Karlsten MD PhD, Uppsala, Sweden

3. Douglas Chamberlain MD, Brighton and Cardiff, UK (advisor during the start-up and manuscript phases only)

4. Johan Herlitz MD, PhD, Borås, Sweden

5. Anna Söderlund RN, Jolife AB, Lund, Sweden (non-voting) (start-up phase-2008)

6. Ulrika Ericsson, RN, Jolife AB, Lund, Sweden (non-voting) (from 2008)

7. Fredrik Arnwald, Director of clinical affairs, Jolife AB, Lund, Sweden (non-voting).

Uppsala Clinical Research Center (UCR), Sweden was appointed CRO to give advice on the outlines of the study, to monitor the study, to give statistical advice, to handle the database, and to perform the statistical analyses of the results. This will be done in accordance with GCP for clinical trials.

### Eligibility criteria for the study

Eligible patients for the LINC-study are adults with an unexpected OHCA for whom an attempt of resuscitation is considered appropriate.

The exclusion criteria are:

1. Traumatic cardiac arrest, including hanging

2. Age believed to be less than 18 years (no upper limit)

3. Known pregnancy

4. Defibrillated before LUCAS™ is brought to the scene

5. Patients body size not fitting the LUCAS™ System

### Study enrolment and randomisation

The device is brought to all patients with dispatch codes for sudden cardiac arrest, loss of consciousness, or other local guidelines suggesting OHCA. Enrolment into the study is at the time that paramedics recognise the situation as a cardiac arrest after they arrive on scene. After confirmation of the arrest, one ambulance professional immediately starts manual CPR while another takes care of the randomisation procedure. All patients fulfilling the inclusion criteria and having no known exclusion criteria are randomised using block randomisation with site (EMS) as stratification variable. Block size is set to 6, but has been kept unknown to all personnel involved throughout the study. Randomisation is at the patient’s side by opening an envelope which is enclosed in the LUCAS™ back pack. Inside the envelope there will be a note prompting either conventional manual or LUCAS™ CPR. A replacement envelope from the block of 6 is added to the LUCAS pack after each use.

**Figure 1:**
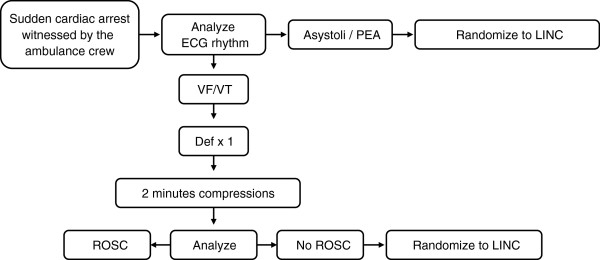
The separate algorithm used for patients suffering a cardiac arrest witnessed by the ambulance crew and used for these witnessed cases for eligible judgment and randomisation into the LINC-study.

Patients suffering a cardiac arrest witnessed by the ambulance crew are handled according to a separate algorithm and if eligible they are randomized into the study (Figure 1).

### Study algorithms and post-resuscitation care

Ventilation and the dosages of drugs in both groups are given according to the International Consensus 2005 as adopted by the European Resuscitation Council [13].

### The LUCAS™ algorithm

**Figure 2:**
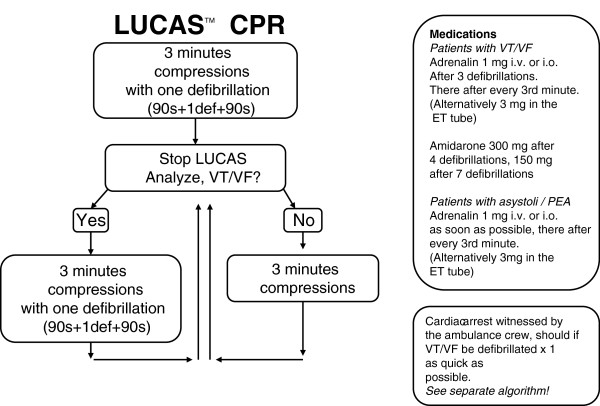
The LUCAS algorithm used in the LINC-study.

Patients randomised to LUCAS™ CPR are treated initially with manual compressions with minimised interruptions until the device is unpacked and ready to use. LUCAS™ is attached to the patient as soon as possible. In this group the defibrillator must be in manual mode. Mechanical compressions are continued initially for 3 min without checking heart rhythm, irrespective of any manual compressions that have been given by bystanders. The sequence is not interrupted for a shock that is delivered during compressions after 90 seconds of this first 3 min cycle, with the remaining 90 seconds of continued compressions to follow. Thus, the first shock is given on the basis only of definite cardiac arrest without knowledge of whether it is or is not ‘shockable’. Subsequently, heart rhythm is determined after each 3 min cycle by interrupting mechanical compressions briefly and never for longer than 10 seconds. If the analysed rhythm is shockable, a new 3 min cycle of compressions is started, incorporating as before one shock delivered after 90 seconds of on-going compressions. If the rhythm is not shockable, a 3 min cycle of compressions without any shock is followed by a new rhythm analysis. Heart rhythm and circulation are checked after each 3 min cycle (Figure 2).

### The conventional manual CPR algorithm

**Figure 3:**
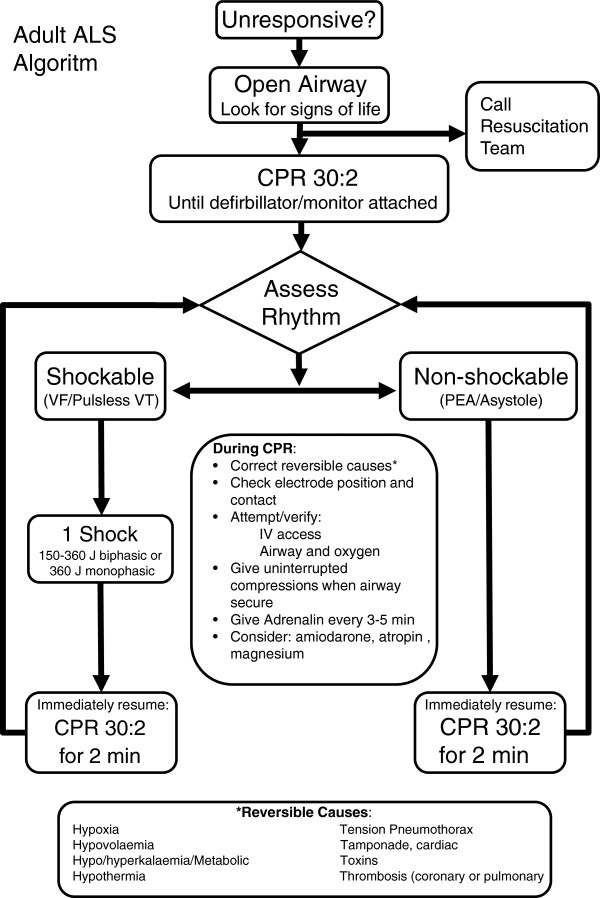
The adult ALS algorithm used as control in the LINC-study.

Those randomised to conventional manual CPR are treated in accordance with the International Consensus 2005 as adopted by the European Resuscitation Council [13] (Figure 3).

### Post resuscitation care

If the patient achieves ROSC, he/she should be treated with mild hypothermia to 32–34 degrees Celsius for 24 h, regardless of initial ECG rhythm, unless contraindications exist. Acute coronary angiography should be considered during the first 48 h of hospital admission.

### Mechanical chest compression devices

Two models of LUCAS have been used during the study. Initially, from January 2008 to early 2010, it was the LUCAS™ 1 Chest Compression System which was a pneumatic gas-driven device. From early 2010, this was replaced by the LUCAS™ 2 Chest Compression System which is powered electrically. Both devices achieve mechanical chest compressions at a constant rate of 100 per minute and to a fixed depth of 4–5 cm by a piston that has a 50% duty cycle, with the added feature of a suction cup that may assist the chest back to neutral position. The LUCAS™ 1 and LUCAS™ 2 devices used in the study both adhere with the 2005 international guidelines on resuscitation [13,14,17,18].

### Patient consent

The nature of the study, in relation to patients who are unconscious, precludes consent before enrolment. It is the responsibility of the investigator to provide, for each surviving subject with mental capacity, full and adequate verbal and written information about the objectives and procedures of the study. They are given the opportunity to ask questions and to decide whether or not they are willing for their results to be included in the dataset. They are also told of their freedom to withdraw from the study at any time and that withdrawal will not affect their future medical care. In cases where the subjects survive without having the mental capacity to accept or withdraw participation, written information is presented to the family, who then take the decision to continue participation or not. Subjects included who do not survive will stay in the study; the family will not be asked for consent. This prevents any positive bias in the results.

### Efficacy and safety evaluation in the study

For efficacy evaluation the following primary and secondary endpoints that will be analysed in the study are:

1. Primary endpoint

a. Four-hour survival after successful restoration of spontaneous circulation (ROSC).

2. Secondary endpoints

a. Restoration of spontaneous circulation defined as a spontaneous palpable pulse.

b. Arrival to the emergency room with spontaneous palpable pulse.

c. Survival to discharge from ICU without severe neurological impairment (CPC 1 or 2).

d. Survival to hospital discharge without severe neurological impairment (CPC 1 or 2).

e. Survival 1 and 6 months after cardiac arrest without severe neurological impairment (CPC 1 or 2).

The safety evaluation in the study comprises three main areas. The first relates to serious adverse events or serious adverse device events. These cover events directly related to CPR, as judged by investigator/co-investigator, and assumed to occur after the randomisation in the study with any of the following sequelae: death; serious deterioration of health in patient; life threatening illness or injury, permanent deterioration of body function or structure; prolongation of hospitalisation; conditions that require medical or surgical treatment to prevent any of the foregoing.

The second area for safety evaluation is any post mortem examination that may reflect injuries from CPR. For comparison of injuries from the two different CPR techniques, autopsies are performed to quantify the number of injuries possibly affecting survival. They are undertaken in three centres (Uppsala, Gävle, and Västerås in Sweden). The aim is to obtain autopsy results from a total of 300 study patients with 150 who have received manual chest compressions only and 150 after LUCAS™ chest compressions.

The third area relates to the criteria for termination of the study stated in the protocol (described above) that would be considered by the steering committee, sponsor, and the independent safety committee within the Scandinavian Society of Anaesthesiology and Intensive Care Medicine research group (SSAI).

### Sample size and statistical methods

Considerations for the sample size calculations are based upon data from the Uppsala-Gävle pilot study, [15] together with data from the National Registry of Cardiac Arrest in Sweden [19]. It was assumed that in the conventional manual treatment group, the proportion of four-hour survival will be 25% and in the LUCAS-CPR treatment group the proportion of four hour survival will be at least 31%. To detect the anticipated difference of at least 6% with a power of 90% in the final analysis, the study requires a total of 2500 patients, i.e. 1250 patients in each treatment group in the intention to treat population. For the intention to treat and predefined populations the primary and all secondary endpoints will be compared between treatment groups with frequency tables and two-sided Fisher’s Exact Tests at the 4.8% and 5% level, respectively; 95% confidence intervals for difference in proportions will be presented where applicable. The result for the primary endpoint in the intention to treat population is confirmative while the other results are regarded as supportive.

Injuries after CPR will be studied in detail. The proportions of patients will be compared between the two treatment groups with frequency tables and two-sided Fisher’s Exact Test at 5% level.

### Analysis populations

The analysis populations are defined as:

Safety population: all randomised patients except surviving patients without informed consent. Intention to treat (ITT) population: all randomised patients except surviving patients without informed consent. Predefined population (PP): all randomised patients, except surviving patients without informed consent, who have completed the study treatment without any protocol violations. Possible protocol violations are:

• Inclusion criterion not fulfilled.

• Primary exclusion criteria subsequently confirmed to have been present.

• Dispatch time to ambulance stop at the address exceeding 12 min.

• Cardiac arrest not witnessed by sight or sound (If data are missing regarding whether the cardiac arrest was witnessed or not, the patient should be regarded as not witnessed).

• LUCAS™ Chest Compression System not brought to the patient or no record form filled in.

If the actual treatment given is not that to which the patient was randomised, the patient will be included in the ITT population set as randomised, but in the PP population according to the actual treatment.

For missing data, the primary endpoint and the first four of the secondary endpoints will be defined according to the worst case principle, i.e. as failures in the ITT population. The confirmative analysis will be performed in the ITT population. The PP analysis will be regarded as supportive. In the PP analysis no imputation of missing values will be performed.

An interim analysis of the primary endpoint was performed during spring 2011 by an independent safety committee within the Scandinavian Society of Anaesthesiology and Intensive Care Medicine research group (SSAI). This was done according to protocol, after 1500 patients had been randomized, using the primary endpoint i.e. 4 h of ROSC, and performed on the intention to treat population. In the interim analysis the criterion for terminating the study is that the p-value from a two-sided Fisher’s Exact Test is less than 0.005. A group-sequential analysis plan [20] is used to preserve a Type I error of at most 5%, so that after completion of the study the primary hypothesis will be tested at a level of 4.8%.

### Data management and source data

The Biometrics section at UCR is responsible for the Data Management and has written a study specific Data Management Plan and a Data Validation Plan. All data is recorded in the Clinical Report Forms (CRFs) and entered via e-CRF directly into a web-based data capturing system. All CRF data is entered into the e-CRF system by the study coordinators at each site. Study data found in either the hospital records or in the paper CRF is considered to be source data. The source data verification ensures consistency between the e-CRF and the paper CRF/patient hospital file.

### Training

Before the study started, all paramedics and other ambulance personnel at each site were trained in conventional manual CPR according to the 2005 guidelines, in the use of LUCAS™ Chest Compressions System, and in the algorithms of the two different treatments [13,14]. The training with the LUCAS™ device was supervised by personnel from the sponsor. Retraining for both techniques continues at least every 6 months during the study period. Personnel involved in the study at the emergency department and at other wards inside the hospital are trained according to the requirements at different sites. Training results for both techniques have been monitored once every year in randomly selected personnel (20%–30%) using a modified CPR manikin (Laerdal, Stavanger, Norway) which measures quality of compressions. This training evaluation has been performed and documented annually from 2009 to 2012.

## Discussion

The LINC-study is the first large multicentre trial powered to evaluate short term survival using mechanical chest compressions with the LUCAS™ device compared with manual chest compressions following conventional guidelines [13,14]. It is being conducted on adult OHCA victims, excluding those who have initial shock success following crew witnessed cardiac arrest or those who have been treated with bystander defibrillation before the arrival of the ambulance service. These groups have a relatively good prognosis. Their exclusion will introduce a negative bias on overall survival in the study, but it will not influence the comparison between the two groups.

The LINC protocol was modified after the Uppsala-Gävle pilot from that originally envisaged in relation to defibrillation strategy [16]. The study as performed uses shocks without analyse during uninterrupted chest compressions for the first sequence in the LUCAS™ group regardless of underlying rhythm, with increased emphasis of having the device on the patient earlier than in the two previous OHCA studies [15,16]. The rationale for having this first shock without analyse in the group treated with LUCAS-CPR is to allow earlier possibility of return of coordinated rhythm in patients with VF/VT. Patients with an initial non-shockable rhythm therefore have one unnecessary shock. The possible harm of this has been extensively discussed within the steering committee after careful evaluation of what is known from the literature, but also after discussion with other experts in the field. The consensus reached has been that the shock without analyse has more potential for benefit than for harm. A further cogent reason for both the initial shock without analyse and for giving a shock halfway through other sequences after detection of a shockable rhythm relates to the known need for the perishock pauses to be a brief as possible [21]. In the LUCAS™ treatment arm, these pauses can be eliminated.

Since no ECG analysis is done before the first defibrillation in the treatment group, a best estimate of the ‘pre-shock ECG rhythm’ is achieved as follows: if a patient has either a pulse or ventricular fibrillation after the first shock, it will be assumed that this patient had ventricular fibrillation before the first shock; if the patient has either asystole or pulseless electrical activity after the first shock, it will be assumed that these patients had the same rhythm before the first shock attempt. ‘Pre-shock’ ECG will be noted in the CRF according to this best estimate.

No other large randomised study using a mechanical chest compression device has been performed or is planned that includes a comparison of injuries from the two different CPR techniques as shown by autopsy [22-24], which is important when placing any new techniques into a clinical context [25]. The safety of the LUCAS™ device has been studied only in small randomised studies and these have not shown any significant differences between manual or mechanical chest compressions despite the concerns that have been raised for this type of treatment [26,27]. A further test of the validity of these results is therefore warranted. It should be noted, however, that even the group randomised to treatment with LUCAS-CPR will have had a period of manual chest compressions before the device was applied, and this could contribute to any injury.

This study has used CPR training intervals similar to those practised in the clinical setting of most EMS in the world, but to ensure quality in both study arms the actual training procedures have been evaluated in randomly selected personnel at each site, as described above.

From spring 2010, the LUCAS™ 1 Chest Compression System was replaced in the study by the LUCAS™ 2 device. This change was made after discussion between the steering committee and the sponsor, and was approved by all of the independent ethics committees. The rationale supporting this decision was that the performance of these two versions of LUCAS™ devices provides mechanical chest compressions with the same rate, depth, duty cycle, and recoil of the chest. This change of device resulted in a few potential benefits for the ambulance crews. They do not need to carry a tank of pressured air, weighing 6.8 kg, which was the power source to LUCAS™ 1. The power source of the LUCAS™ 2 is instead an integrated battery with a weight of 0.6 kg. Secondly, the LUCAS™ 2 is also able to perform chest compressions in 30:2 mode.

During the spring of 2011 the planned interim analysis of the LINC-study was carried out by the Scandinavian Society of Anaesthesiology and Intensive Care Medicine research group. The results of the analysis allowed the study to continue, a decision endorsed by the LINC steering committee. This means that the enrolment continued and the final patient was enrolled in the end of August, 2012, with complete outcome data available 6 month later. The results are expected to be published in a scientific journal during 2013.

## Conclusion

The LINC-study was designed to evaluate the efficacy and safety of a resuscitation method for OHCA using mechanical chest compressions with the LUCAS™ device associated with defibrillation during compression and to compare the results with those of manual CPR according to 2005 guidelines. As a large randomised multicentre study, it will contribute to knowledge of OHCA and to the role of the mechanical chest compression with LUCAS™.

## Competing interest

Two employees (BMH and FA) from Physio-Control/Jolife AB participated in the outline and writing of this manuscript. Sten Rubertsson has received consultation fee from Physio-Control/Jolife AB. All the other authors’ institutions receive funding from Physio-Control/Jolife AB as a compensation for the work with the patient inclusions in the study except for that of DC who was not actively involved during the time of the study.

## Authors’ contribution

SR is the principal investigator for the trial. The trial protocol was developed by; SR, JH, RK, DC and AS. BMH, SR and DC prepared the first draft of this summary protocol manuscript and revised in the light of comments from all authors. All authors have approved this final version of the manuscript. After finalising the protocol, it was endorsed by the sponsoring company.

## Role of the funding source

Physio-Control/Jolife AB is funding the LINC-study. Statistical consultation was done with statisticians from the UCR.
